# Real-time 3-T MRI of peroneal tendon motion during active ankle movement: feasibility and image quality

**DOI:** 10.1186/s41747-026-00762-7

**Published:** 2026-06-25

**Authors:** Dan Mocanu, Chalermkwan Noinar Phoemsawang, Evin Ina Papalini, Katarzyna Bokwa-Dąbrowska, Katarina Nilsson Helander, Isabella M. Björkman-Burtscher, Pawel Szaro

**Affiliations:** 1 Department of Radiology, Institute of Clinical Sciences, Sahlgrenska Academy, University of Gothenburg, Gothenburg, Sweden; 2 Department of Radiology, Sahlgrenska University Hospital, Region Västra Götaland, Gothenburg, Sweden; 3 Department of Biomedical Engineering and Medical Physics, Sahlgrenska University Hospital, Gothenburg, Sweden; 4 Department of Medical Radiation Sciences, Institute of Clinical Sciences, Sahlgrenska Academy, University of Gothenburg, Gothenburg, Sweden; 5 Department of Orthopedics, Institute of Clinical Sciences, Sahlgrenska Academy, University of Gothenburg, Gothenburg, Sweden; 6 Department of Orthopedics, Sahlgrenska University Hospital, Region Västra Götaland, Gothenburg, Sweden

**Keywords:** Ankle joint, Joint instability, Magnetic resonance imaging, Tendons, Volunteers

## Abstract

**Objective:**

To evaluate the feasibility and image quality of dynamic 3-T magnetic resonance imaging (MRI) for visualizing and quantifying peroneal tendon motion during active dorsiflexion and plantarflexion.

**Materials and methods:**

This prospective pilot study was approved by the Swedish Ethical Review Authority (Dnr 2020-00029). Asymptomatic adults without prior ankle trauma underwent dynamic ankle MRI at 3 T using a balanced fast field-echo sequence (axial at the lateral malleolus) during active dorsiflexion/plantarflexion. Three musculoskeletal radiologists rated five image-quality criteria (five-point Likert); agreement was quantified with Gwet’s AC2 and Fleiss’ κ. The highest-rated sequence was analyzed. One musculoskeletal radiologist manually segmented the peroneus longus and peroneus brevis tendons; Euclidean centroid distance was computed per frame. For intra-rater analysis, an independent person randomly selected three volunteers; segmentation was repeated after 1 month, and reliability was assessed with the intraclass correlation coefficient (ICC) (2,1).

**Results:**

Ten volunteers aged 36.6 ± 7.8 years (mean ± stasndard deviation) were included. Tendon motion was visualized in all volunteers with good image quality (median 4; mean 4.04–4.10). Agreement was strongest for retinaculum visibility and ability to follow tendon motion (AC2 = 0.621 and 0.557, respectively). Centroid distance increased in dorsiflexion and decreased in plantarflexion (excursion 2.95–4.83 mm). Intra-rater reliability was good (ICC = 0.85; 95% confidence interval 0.723–0.922; *p* < 0.001).

**Conclusion:**

Dynamic MRI enabled reproducible, high-quality visualization and quantification of peroneal tendon motion during active ankle movement and warrants evaluation in suspected peroneal tendon instability.

**Relevance statement:**

Dynamic real-time 3-T MRI enables visualization and quantitative tracking of peroneal tendon motion during active ankle movement, and may complement static imaging in future studies of suspected peroneal tendon instability.

**Key Points:**

- Real-time 3-T MRI showed peroneal tendon motion in all volunteers.
- Image quality was consistently good for tracking tendons during ankle movement.
- Centroid-to-centroid distance between tendons was highly repeatable over time.

**Graphical Abstract:**

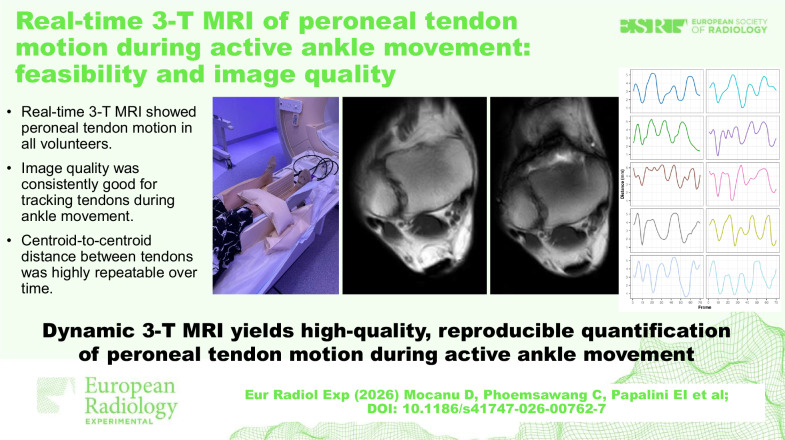

## Background

Peroneal pathology is a frequent, under-recognized cause of lateral ankle pain. Anterior talofibular ligament and calcaneofibular ligament injuries predispose to peroneus brevis (PB) tears and lateral instability, and the superior peroneal retinaculum may be concomitantly injured [1, 2]. Peroneal tendon instability and longitudinal split tears can be challenging to diagnose on clinical examination and routine static imaging. False-negative findings may occur when instability is intermittent or not reproduced during testing, when imaging is performed without dynamic assessment, and because MRI sensitivity for split tears is imperfect [3]. Accurate diagnosis requires both functional evaluation and detailed visualization of thin soft-tissue structures. However, few clinical imaging tools are available for functional tendon assessment, and there is no consensus on the preferred imaging modality after acute or chronic ankle injury [4].

Dynamic visualization permits direct evaluation of peroneal tendon stability and motion during active movement, revealing behavior that static techniques do not capture. Nevertheless, an objective and reproducible method for standardized, motion-dependent stability assessment is lacking. Ultrasound enables real-time interrogation of ankle structures, but its diagnostic performance depends heavily on examiner expertise, limiting reproducibility, interobserver reliability, and retrospective review [5]. Studies performed by expert operators may also overestimate performance relative to routine practice, and large ankle-specific datasets are scarce. Conventional MRI is widely used, but its diagnostic utility for chronic ligament injuries may be lower than that of ultrasound due to static acquisitions. Several studies have demonstrated the feasibility of dynamic MRI in assessing knee joint motion, temporomandibular joint motion, thoracic wall motion, and large-joint kinematics using passive motion devices, albeit with insufficient image resolution for small ankle structures. The few ankle studies to date have addressed joint kinematics with phase-contrast imaging and positioning devices or muscle moment arms with ultrafast low-resolution imaging, rather than high-resolution tendon behavior. No study has focused on dynamic, high-resolution visualization of the peroneal tendons during active ankle motion at 3T [6].

Clinically, intermittent peroneal tendon instability may be motion- and position-dependent and therefore may not be consistently captured on routine static MRI. Dynamic ultrasound is widely used as the first-line modality for suspected peroneal tendon instability and allows real-time interrogation of tendon motion; however, it is operator dependent, and findings may be difficult to standardize or review retrospectively. Ultrasound is the preferred first-line test for suspected peroneal instability, although reported accuracy estimates are based on small, selected cohorts [7–9]. In this context, a dynamic 3-T MRI add-on may provide a standardized, cross-sectional, and reviewable functional assessment of the peroneal tendons and adjacent stabilizing structures during active dorsiflexion and plantarflexion within the same examination as conventional morphological ankle MRI, complementing rather than replacing ultrasound and static MRI.

We hypothesized that a dedicated dynamic 3-T MRI approach can feasibly depict peroneal tendon motion with image quality sufficient for quantitative analysis. In this prospective pilot study, we evaluated image quality and assessed the feasibility of a two-dimensional dynamic MRI sequence for visualizing and quantifying peroneal tendon motion at the lateral malleolus during active dorsiflexion and plantarflexion in healthy volunteers. We obtained structured image-quality ratings from musculoskeletal radiologists and quantified tendon separation via centroid-distance metrics with reliability testing. By optimizing a high-resolution, temporally-resolved MRI sequence, we aimed to address the current diagnostic gap and lay the groundwork for a reproducible, clinically applicable method for functional assessment of peroneal tendon stability. This pilot study focuses on feasibility and measurement prerequisites.

## Methods

### Study design and ethics

This prospective, cross-sectional observational pilot study in healthy volunteers was approved by the Swedish Ethical Review Authority (Dnr 2020-00029, approved 2020-05-26). Written informed consent was obtained from all volunteers in accordance with the Declaration of Helsinki.

### Setting

Imaging was performed at the Department of Radiology, Sahlgrenska University Hospital, Gothenburg, Sweden, between September 2024 and January 2025. Volunteer recruitment and data collection were conducted during the same period. Volunteer inclusion and the workflow for image-quality rating and quantitative analysis are summarized in the study flow diagram (Supplementary Fig. S1).

### Volunteers

Ten healthy adult volunteers were prospectively included (5 women, 5 men; age range 23–48 years; mean 36.6 ± 7.8 years). Inclusion criteria were: age ≥ 18 years, no history of ankle trauma or surgery, no ankle pain, and no MRI contraindications. Exclusion criteria were: any known ankle pain or prior ankle pathology, metal implants, or the inability to perform active ankle motion. Volunteers were recruited from hospital staff. The same dynamic sequence was acquired four times in each examination.

#### Instructions to volunteers and MRI protocol

Prior to imaging, all volunteers received standardized instructions from a radiographer to ensure consistent and controlled ankle movements. Slow dorsiflexion and plantarflexion were demonstrated through manual guidance, enabling volunteers to adopt a consistent rhythm. Foot movement was monitored in real-time using a patient-monitoring camera, allowing immediate adjustments to movement rhythm and execution during scanning.

**Fig. 1:**
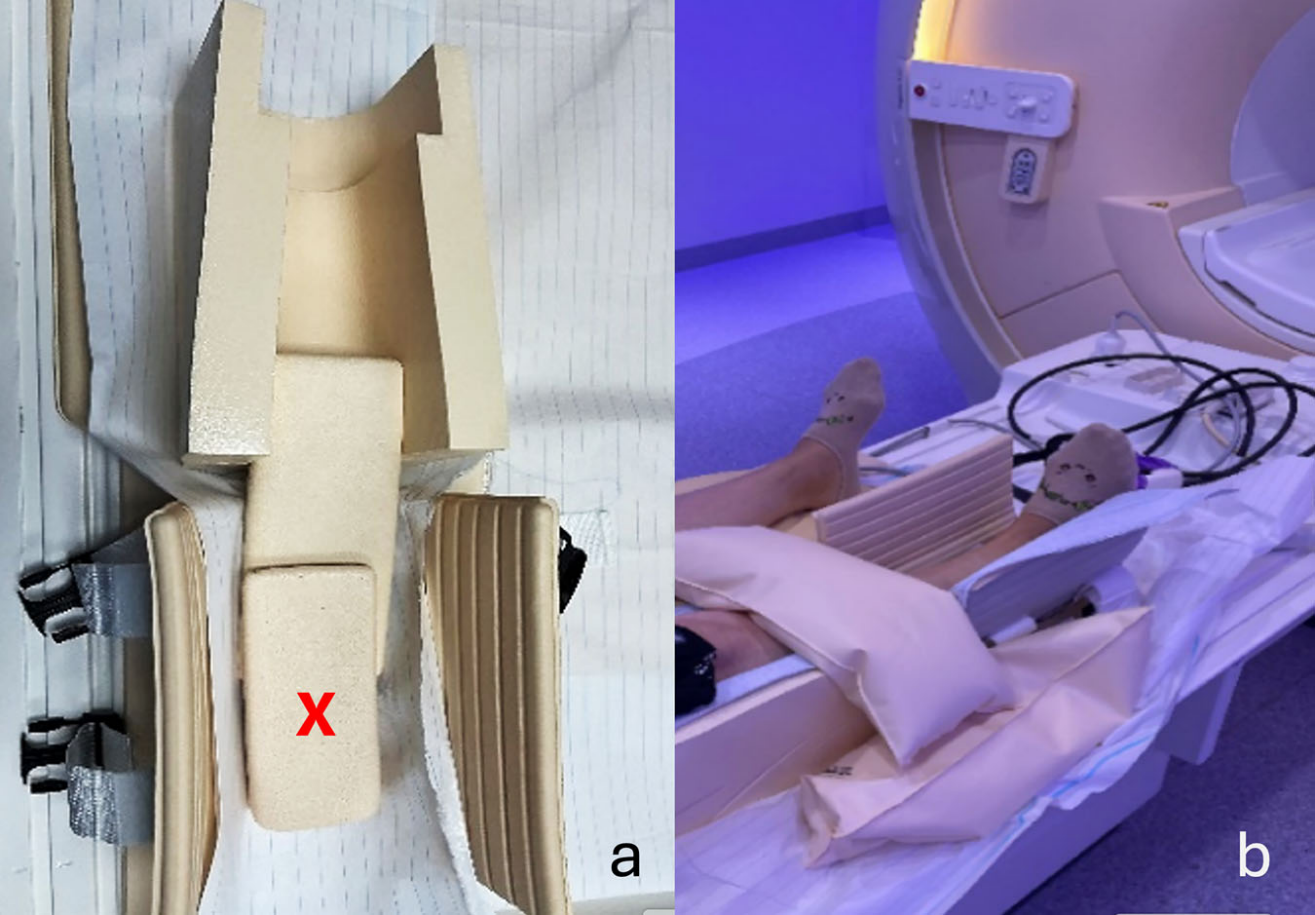
Set up for dynamic MRI of the ankle. **a** Coil and cushion arrangement; cushion support elevates the lower leg; “X” indicates the position of the ankle joint, placed distal to the center of the coil. **b** Volunteer in the scanning position; sandbags were placed on and beside the imaged lower leg for stabilization

Examinations were performed on a 3-T scanner (Ingenia, Philips Medical Systems) and a 16-channel small extremity coil. Volunteers were positioned supine with the ankle joint placed distal to the coil’s center, allowing the toes to extend outside the coil to maximize the range of motion during dorsiflexion and plantarflexion (Fig. 1). The flexible coil elements were kept slightly separated to facilitate unrestricted foot movement. The lower leg and ankle were elevated and stabilized using cushions and sandbags to isolate ankle flexion and reduce unwanted motion in adjacent structures. Sandbags were placed proximal to the ankle, ending above the ankle joint, to limit lower-leg motion without restricting the ankle joint, which needed to move freely during the dynamic task.

**Fig. 2:**
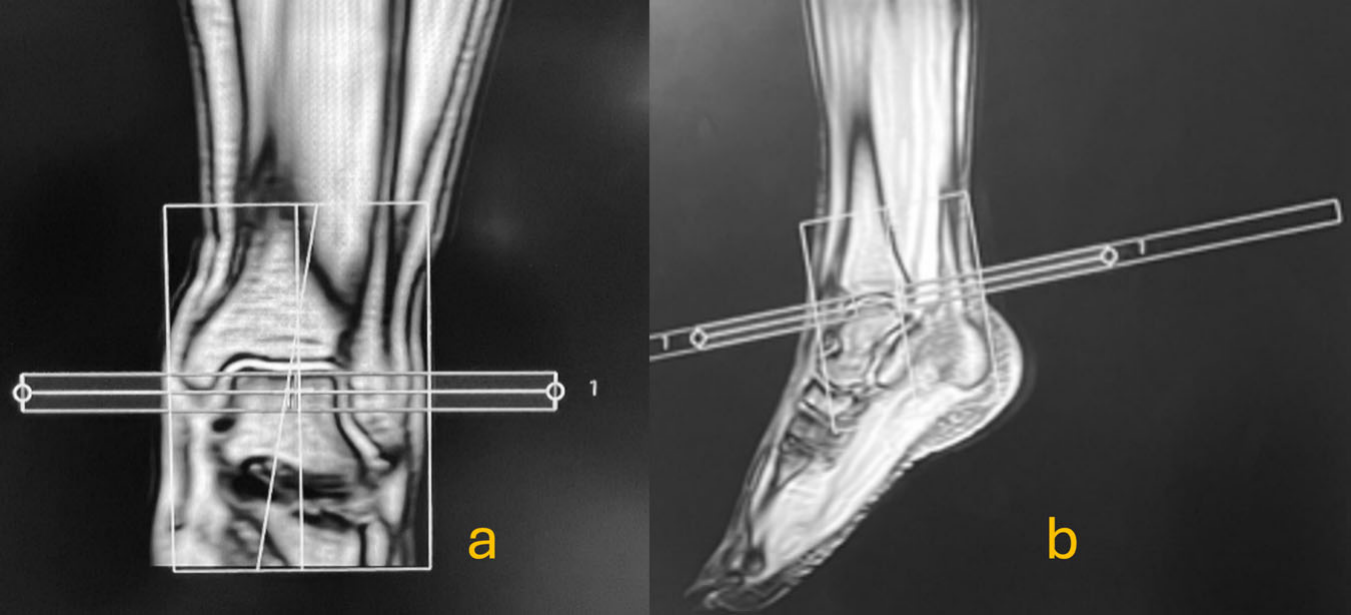
Planning of the transverse imaging plane for the dynamic ankle sequence. The plane was aligned parallel to the distal tibial articular surface and positioned to intersect the ankle joint space, as defined on coronal (**a**) and sagittal (**b**) localizer images

Dynamic imaging was acquired in the axial plane at the level of the lateral malleolus, a common site for peroneal tendon instability, dislocation, and split tears, and a frequent target in surgical repair. The transverse plane for the dynamic sequence was aligned parallel to the distal tibial articular surface and positioned to intersect the ankle joint space (Fig. 2).

A balanced fast field echo sequence was selected and optimized to achieve high soft‑tissue contrast and temporal resolution (repetition time/echo time = 4.5/2.2 ms; flip angle = 45°; field of view = 140 × 200 mm²; acquisition matrix = 200 × 284; half scan = 0.625; number of excitations = 1; acquired in‑plane resolution = 0.70 × 0.70 mm²; reconstructed in‑plane resolution = 0.39 × 0.39 mm²; slice thickness = 10 mm; compressed sensitivity encoding acceleration factor = 3.1; temporal resolution = 0.59 s/frame; total scan time = 60 s). Each dynamic series comprised 70 frames, and the same dynamic balanced fast field echo sequence was repeated four times during a single imaging session.

In addition to the standard morphological ankle MRI protocol, the four dynamic sequences required brief patient instruction and positioning for active dorsiflexion/plantarflexion; the overall additional time (set-up/repositioning plus acquisition) was approximately 8 min.

### Image quality assessment

Image quality of the dynamic sequences was the primary outcome. Image quality was independently evaluated by three musculoskeletal radiologists (D.M., P.S., and K.B.D. with 14, 10, and 8 years of experience in musculoskeletal radiology, respectively) using a five-point Likert scale based on five predefined criteria (Table 1): (1) signal-to-noise ratio (SNR); (2) contour visibility of the peroneal tendons; (3) visualization of the fibula and superior peroneal retinaculum; (4) depiction of tendon motion throughout the dynamic sequence; and (5) diagnostic confidence in excluding peroneal tendon luxation. In addition to numerical scoring, raters could provide optional free-text comments regarding image quality and the presence of artifacts potentially interfering with diagnostic evaluation (6). Sequences were deidentified and presented in randomized order, with raters blinded to volunteer identity, sequence parameters, and acquisition order. Finally, raters were asked to indicate which of the four dynamic sequences they subjectively considered most suitable for clinical reporting and tendon analyses (7) (Table 1). The sequence with the highest overall diagnostic quality was selected for further analysis. Radiologists received standardized instructions, and scoring guidelines were established prior to rating.

**Table 1:** Image quality assessment criteria and Likert scale score definitions

Image quality parameter	Likert scale score	Definition of score
1. SNR and presence of artifacts	5	Absence of artifacts
	4	Minor artifacts without diagnostic impact
	3	Moderate artifacts with preserved diagnostic utility
	2	Severe artifacts obscuring tendon visibility
	1	Only artifacts visible; sequence non-diagnostic
2. Contour visibility of the peroneal tendons	5	Sharply defined and continuous outlines
	4	Slight blurring of contours
	3	Partial visibility of contours
	2	Poorly defined contours with retained tendon visibility
	1	Tendons are indistinct or non-discernible
3. Visualization of the fibula and superior peroneal retinaculum	5	Both structures are clearly visualized
	4	One structure is slightly blurred; the other is clearly visible
	3	One structure is clearly visible; the other is partially visible
	2	Both structures are poorly visible
	1	Neither structure is adequately visible
4. Depiction of tendon motion throughout the dynamic sequence	5	Smooth and continuous motion across the entire sequence
	4	Motion visible in > 50% of frames
	3	Motion visible in < 50% of frames
	2	Indistinct tendon movement
	1	No clear motion is identifiable
5. Diagnostic confidence in excluding peroneal tendon luxation	5–1	Subjective score reflecting the rater’s confidence in ruling out dislocation based on image quality
6. Free text comments (optional)		
7. Please indicate the sequence you consider most suitable for clinical reporting and tendon analysis by selecting one sequence based on your overall subjective assessment	Sequences 1, 2, 3, or 4	

### Tendon motion assessment

Tendon motion was the secondary outcome and was defined as the frame-by-frame Euclidean distance between the centroids of the PB and peroneus longus (PL) tendons during active movement. This quantitative distance was calculated from manually segmented contours in each frame and used to evaluate temporal tendon motion. The centroid was defined as the geometric center of each segmented tendon.

For each volunteer, the series with the highest total image-quality score (sum of all reader ratings across the five image-quality criteria) was selected for analysis. Images were manually segmented by a musculoskeletal radiologist with 10 years of experience (P.S.), using a custom ImageJ macro (version 2.16.0/1.54 g). For the intrarater agreement analysis, three of the ten volunteers were randomly selected by an independent person not involved in the study. The rater was blinded to previous segmentations and volunteer identity during the second session.

Manual segmentation was performed for the fibula and the PL and PB tendons (Supplementary material 1, Code A). The macro extracted and exported the *X*/*Y* coordinates of each structure into structured text files. A script (Supplementary material 1, Code B) parsed the coordinate data, identified the centroids of each tendon, and merged the values by volunteer and time point. For each segmented tendon, the centroid was calculated based on the spatial distribution of all pixels within the manually delineated contour.

### Motion curve analysis

To quantify tendon motion, the Euclidean distance between the centroids of the PB and PL tendons was calculated for each frame of the dynamic sequence. The distance was computed using the formula below, where (*x*_PB_, *y*_PB_) and (*x*_PL_, *y*_PL_) represent the centroid coordinates of the PB and PL, respectively:

$$d=\sqrt{\left\{{\left({x}_{\left\{{PB}\right\}}-{x}_{\left\{{PL}\right\}}\right)}^{2}+{\left({y}_{\left\{{PB}\right\}}-{y}_{\left\{{PL}\right\}}\right)}^{2}\right\}}$$

Euclidean distances between the centroids of the PB and PL tendons were calculated (Supplementary material 1, Code B). Distances were exported for analysis and visualization. These distances were used to generate motion curves reflecting tendon motion over time. Centroid-to-centroid distance between the PB and PL tendons was used as a reproducible quantitative descriptor of their relative spatial relationship over time, reflecting inter-tendon spacing and translation during motion.

For visualization, motion curves were smoothed using locally estimated scatterplot smoothing–LOESS as a function of normalized time (0–1). Smoothing was performed separately for each volunteer in R using loess with a span of 0.2. The smoothed curves were used for visualization only; quantitative analyses were based on the raw, unsmoothed data.

### Sample size and statistical analysis

Due to a lack of similar studies, the effect size is not known. As this was a pilot feasibility study, a formal sample size calculation was not performed. The findings were intended to inform the design of future powered diagnostic studies. Analyses of tendon-motion patterns beyond intra-rater reliability were exploratory and descriptive, because this pilot feasibility study was not powered for formal inferential comparisons between volunteers or motion phases.

Although image quality was rated on a five-point Likert scale (ordinal variable), we treated the scores as continuous for analysis, consistent with common practice. This approach is supported when distributions approximate interval behavior, as in our case, confirmed by histograms and reporting of both mean and median scores. We followed previous guidance, which notes that Likert data can be appropriately analyzed in this manner [10, 11].

To assess inter-rater agreement for image quality ratings, we used both Fleiss κ and Gwet’s agreement coefficient 2 (AC2), reporting confidence intervals (CIs), *z*-values, and *p*-values. Gwet’s AC2 was included to account for the known paradoxical behavior of Fleiss κ in high-agreement scenarios. The agreement was interpreted using conventional benchmarks [12, 13].

Descriptive statistics were used to summarize volunteer characteristics and centroid distances between the PL and PB tendons. For each volunteer, we calculated the minimum, maximum, median, mean, standard deviation (SD), and excursion (range between maximum and minimum) of tendon centroid distances across time frames. The proportion of frames with centroid distances below 1.5 mm and above 4 mm was also computed. Skewness was calculated to describe asymmetry in tendon separation patterns. Normality of distributions was assessed using the Shapiro-Wilk test. Intra-rater reliability for centroid distances was evaluated using the intraclass correlation coefficient (ICC) with a two-way random-effects model for single measurements and absolute agreement, *i.e.*, ICC (2,1), following the guidelines of Koo and Li [12]. Ninety-five per cent CIs (95% CIs) and F-statistics were reported.

All statistical analyses were performed in RStudio (version 4.4.3), using the *irr* and *rel* packages for reliability analysis and base R functions for descriptive summaries and inferential tests. All plots were created using R (*ggplot2* and *ComplexHeatmap* packages).

## Results

### Image quality assessment

**Fig. 3:**
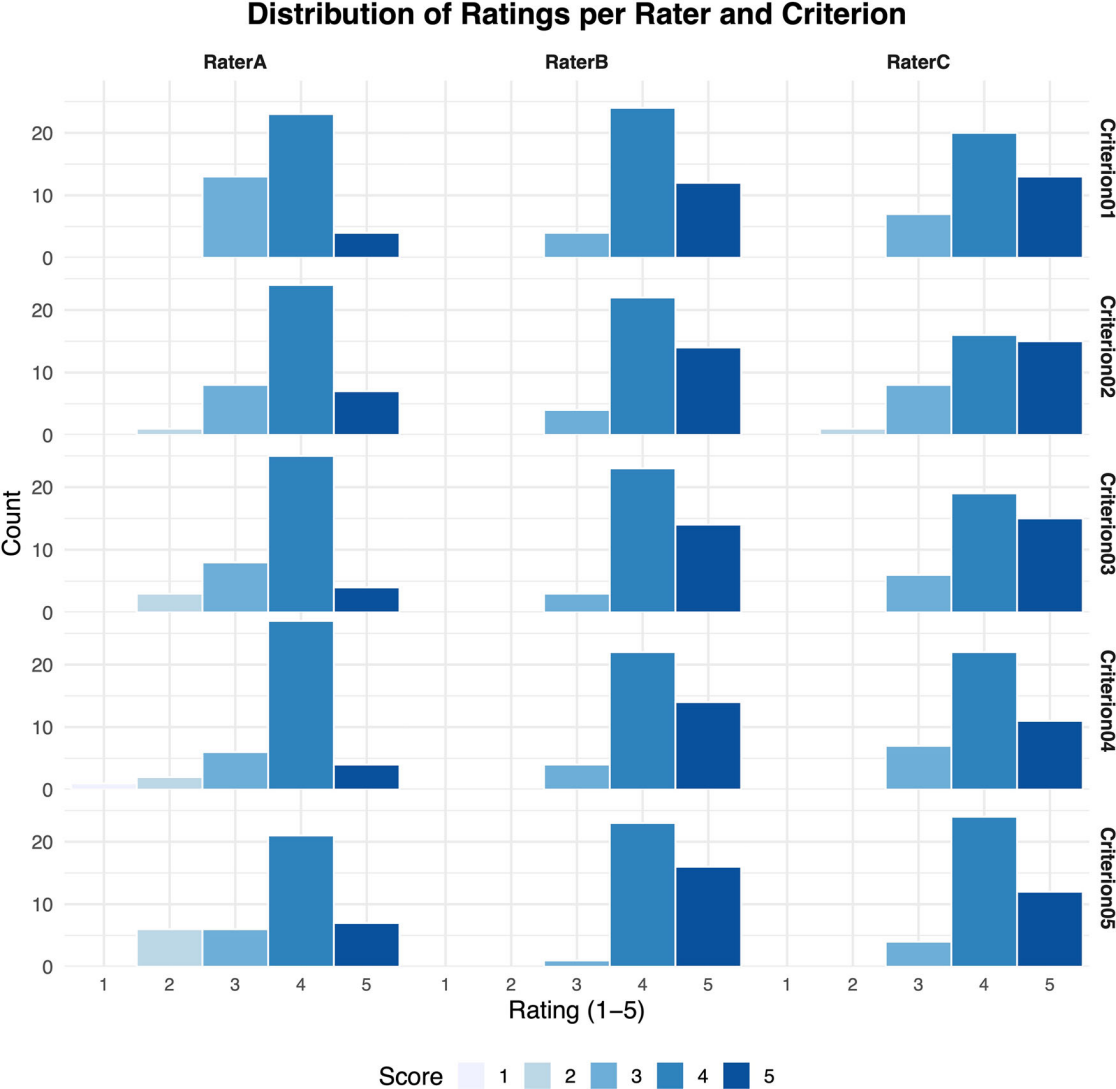
Distribution of scores per rater and criterion on a 5-point Likert scale. Histograms illustrate the frequency of scores assigned by each rater for each evaluation criterion. Criteria: 1- SNR and artifacts; 2- Sharpness of peroneal tendon contours; 3- Visibility of anatomical landmarks in direct relation to the peroneus tendons (fibula and retinaculum); 4- Ability to follow tendon motion; and 5- Diagnostic confidence, *i.e*., level of confidence in evaluating tendon function based on the image quality

Across sequences, diagnostic confidence (criterion 5) and sharpness of peroneal tendon contours (criterion 2) received the highest total ratings (*n* = 120, sum = 492, mean = 4.10), while diagnostic confidence (criterion 5) showed the greatest variability in ratings (SD = 0.76), indicating somewhat less agreement among readers despite high overall scores (Table 2 and Fig. 3). Median scores were ≥ 4.0 for all five criteria, indicative of good image quality.

**Table 2:** Image quality evaluation results

Likert scale criterion	Total scores	Mean	Median	SD	Range	N scores for Likert score				
						1	2	3	4	5
1: SNR and artifacts	485	4.04	4	0.67	3–5	0	0	24	67	29
2: Sharpness of peroneal tendon contours	492	4.1	4	0.73	2–5	0	2	20	62	36
3: Visibility of anatomical landmarks in direct relation to the peroneus tendons (fibula and retinaculum)	490	4.08	4	0.72	2–5	0	3	17	67	33
4: Ability to follow tendon motion	485	4.04	4	0.73	1–5	1	2	17	71	29
5: Diagnostic confidence - level of confidence in evaluating tendon function based on the image quality	492	4.1	4	0.76	2–5	0	6	11	68	35

*SD* Standard deviation

The agreement in quality assessment was moderate using Gwet’s AC2 and fair-to-moderate using Fleiss κ (Table 3). Agreement was highest for the visibility of the superior peroneal retinaculum and the ability to follow peroneal tendon motion.

**Table 3:** Agreement between raters for image quality

Criterion	Fleiss’ κ	κ CI	κ Z	κ *p*	Gwet’s AC2	AC2 CI	AC2 Z	AC2 *p*
1	0.347	[0.229, 0.465]	6.17	6.829 × 10^-10^	0.564	[0.529, 0.603]	30.98	1.003 × 10^-210^
2	0.272	[0.147, 0.397]	4.79	1.668 × 10^-6^	0.515	[0.473, 0.556]	24.21	1.746 × 10^-129^
3	0.431	[0.321, 0.541]	7.52	5.478 × 10^-14^	0.621	[0.589, 0.653]	38.12	5.975 × 10^-318^
4	0.335	[0.216, 0.454]	5.87	4.358 × 10^-9^	0.557	[0.518, 0.595]	28.41	1.522 × 10^-177^
5	0.301	[0.179, 0.423]	5.05	4.418 × 10^-7^	0.534	[0.496, 0.572]	27.24	2.183 × 10^-163^

Criterion: 1—SNR and artifacts. 2—Sharpness of peroneal tendon contours. 3—Visibility of anatomical landmarks in direct relation to the peroneus tendons (fibula and retinaculum). 4—Ability to follow tendon motion. 5—Diagnostic confidence, *i.e.*, level of confidence in evaluating tendon function based on the image quality

*κ* Fleiss’ kappa, *CI* Confidence interval, *Z* *Z* statistic, *p* *p* value, *AC2* Gwet’s agreement coefficient 2, *CI values* 95% Confidence intervals

Optional free-text comments were infrequent and mainly noted occasional motion-related blurring/ghosting or through-plane/partial-volume effects affecting tendon contour sharpness; no rater reported artifacts that precluded diagnostic interpretation.

### Visualization of ankle and tendon movement

**Fig. 4:**
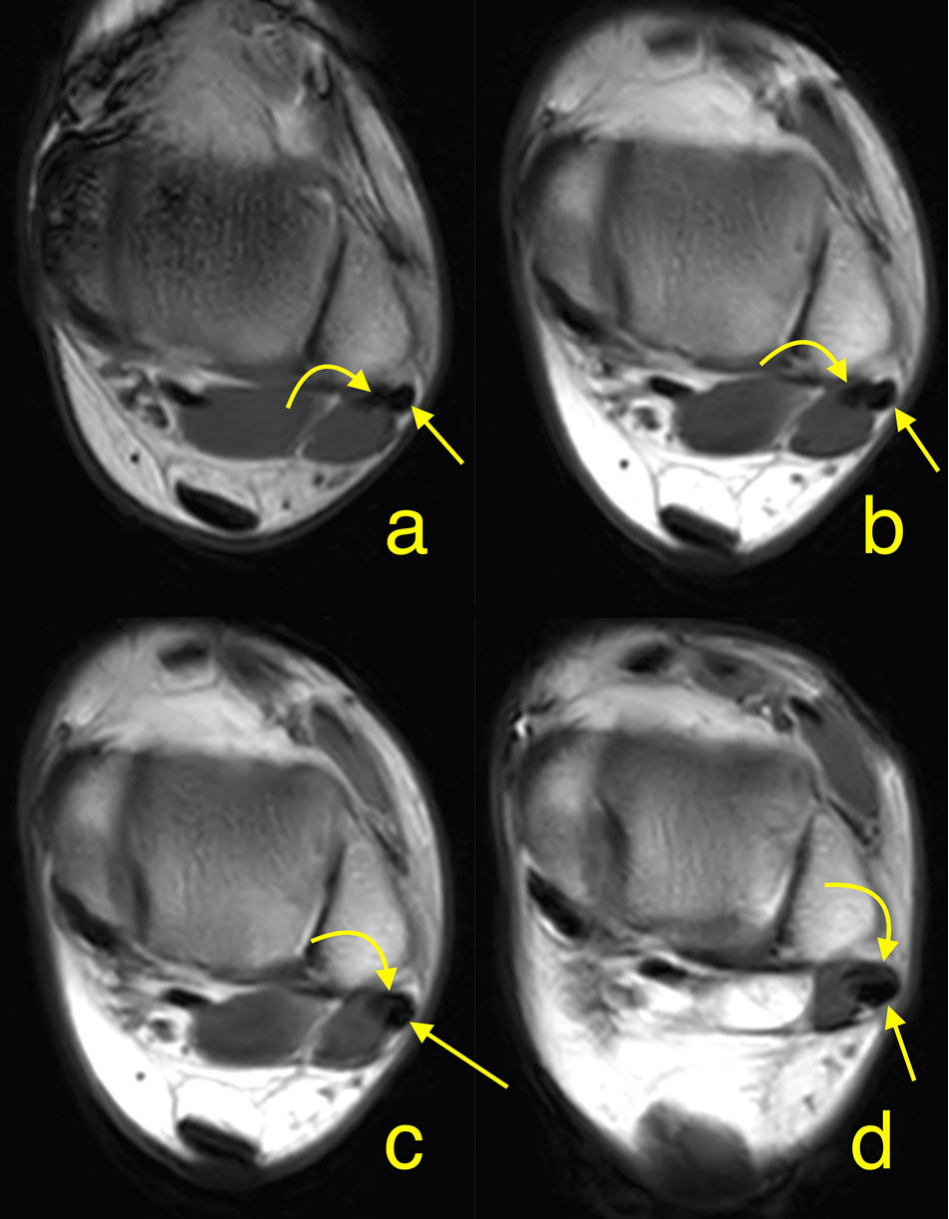
Dynamic 3-T MRI of the ankle at the level of the lateral malleolus, transverse sections. **a**–**d** Show sequential frames used to assess peroneal tendon stability, from plantarflexion (**a**) to dorsiflexion (**d**). The PB is indicated by a curved arrow, and the PL by a straight arrow

Volunteers successfully performed continuous self-paced plantar-/dorsiflexion during all acquisitions, completing 4–5 cycles per 60-s cine scan under real-time camera monitoring. Movement of the peroneal tendons was visualized in all volunteers (Fig. 4, Supplementary material: Supplementary Videos 1 and 2).

Differences between tendons during plantarflexion and dorsiflexion

We observed good intra-rater reliability for the distance between centroids of the peroneus tendons. The ICC value was 0.85 (95% CI: 0.723–0.922), with a statistically significant result (F(33, 33.98) = 12.43, *p* = 9.385 × 10^-12^).

**Fig. 5:**
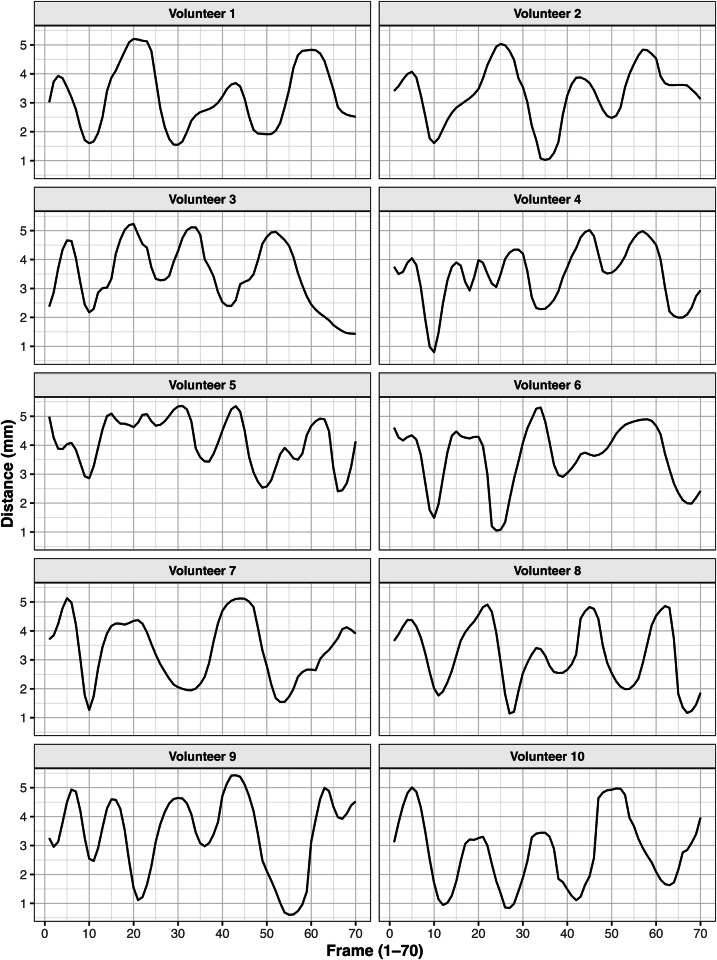
LOESS-smoothed dynamic MRI-derived distance curves between the centroids of the PL and PB tendons during active ankle motion in ten healthy volunteers. The *y*-axis shows the distance (mm) between tendon centroids, and the *x*-axis shows the frame number (1–70). Smoothing was performed separately for each volunteer using LOESS with a span of 0.2. The smoothed curves are shown for visualization only. LOESS, Locally estimated scatterplot smoothing; MRI, Magnetic resonance imaging

**Fig. 6:**
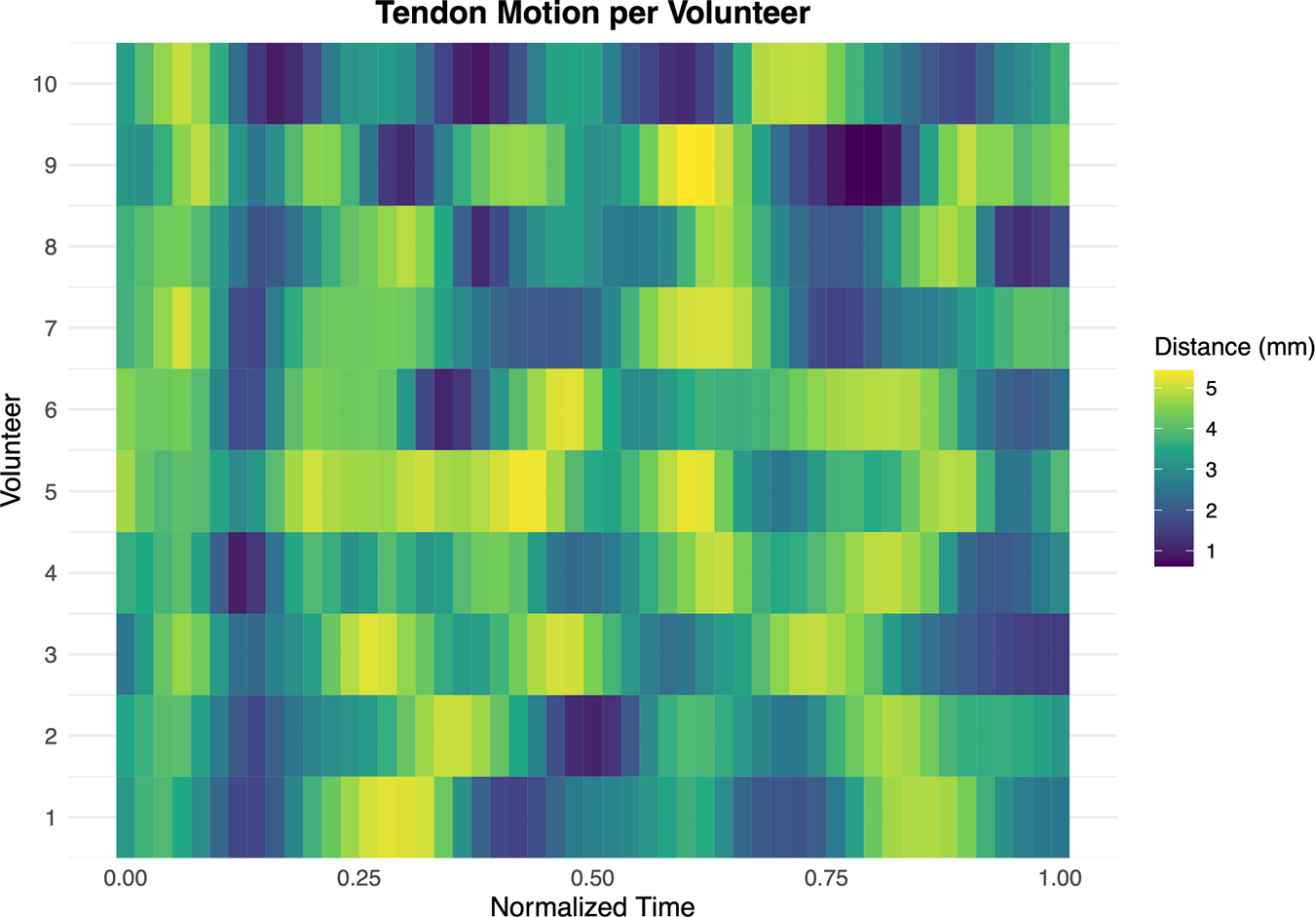
Heatmap of centroid distances between the PL and PB tendons over time for each volunteer. Each row represents one volunteer, and each column corresponds to a time point during the 60-s dynamic magnetic resonance imaging sequence. Color intensity reflects the magnitude of tendon separation (distance in mm)

The distance between the tendons’ centroids increased in dorsiflexion and decreased in plantarflexion. Quantitative analysis of the centroid distances revealed volunteer-specific patterns of tendon motion (Table 4 and Figs. 4–6). The minimum tendon-to-tendon distances ranged from 0.61 mm to 2.40 mm in plantarflexion, and maximum distances ranged from 4.91 mm to 5.44 mm in dorsiflexion (Table 4). Normality testing (Shapiro–Wilk) indicated that the distributions were not normally distributed in any of the volunteers, with exact *p*-values ranging from *p* = 2.097 × 10⁻¹⁰ to *p* = 8.055 × 10⁻⁷ across volunteers.

**Table 4:** Summary of peroneal tendon motion metrics based on centroid distance curves for each volunteer. Excursion represents the difference between the maximum and minimum values

Volunteer	Min (mm)	Max (mm)	Excursion (mm)	Median (mm)	Mean (mm)	SD (mm)	% Frames < 1.5 mm	% Frames > 4 mm	Skewness	*p* value (Shapiro–Wilk)
1	1.54	5.21	3.67	3.12	3.20	0.99	0.00	22.22	0.27	*p* = 3.55 × 10⁻⁵
2	1.02	5.04	4.02	3.38	3.22	0.99	5.51	20.76	-0.30	*p* = 2.30 × 10⁻⁹
3	1.43	5.24	3.81	3.64	3.61	0.95	2.40	38.80	-0.30	*p* = 2.81 × 10⁻⁵
4	0.76	5.03	4.26	3.48	3.29	0.97	5.76	22.63	-0.47	*p* = 2.10 × 10⁻¹⁰
5	2.40	5.36	2.95	4.00	4.03	0.78	0.00	50.21	-0.20	*p* = 1.29 × 10⁻⁵
6	1.04	5.34	4.31	3.59	3.38	1.09	5.71	35.10	-0.32	*p* = 1.50 × 10⁻⁸
7	1.26	5.16	3.90	3.35	3.31	1.05	2.08	32.08	-0.05	*p* = 8.05 × 10⁻⁷
8	1.08	4.91	3.83	3.14	3.13	1.03	6.94	24.90	-0.09	*p* = 2.50 × 10⁻⁵
9	0.61	5.44	4.83	3.50	3.36	1.19	10.20	35.10	-0.50	*p* = 1.39 × 10⁻⁵
10	0.81	5.01	4.20	2.78	2.79	1.13	14.69	17.55	0.22	*p* = 2.66 × 10⁻⁶

Tendon-motion patterns varied between volunteers (Supplementary Videos 1 and 2, Figs. 5 and 6, and Supplementary Fig. S2). Visual inspection suggested inter-individual variation in motion-curve shape and amplitude (Supplementary Material 2, Fig. S3a–c). Volunteers 3 and 9 showed smaller or more irregular amplitudes (Fig. S3a), volunteer 7 showed sharper peaks in the speed plot (Fig. S3b), and volunteers 4 and 10 showed more abrupt changes in the curvature plot (Fig. S3ac).

## Discussion

This prospective study demonstrates the technical feasibility of dynamic 3-T MRI to evaluate peroneal tendon motion during active dorsiflexion and plantarflexion with good image quality in healthy volunteers. Image-quality ratings were high across all five evaluation criteria (median 4), and inter-reader agreement for these criteria was moderate-to-substantial when quantified with Gwet’s AC2. Quantitative analysis of centroid distances showed the expected directional changes, namely an increase in dorsiflexion and a decrease in plantarflexion, with excursions ranging from 2.95–4.83 mm, and good intra-rater reliability (ICC(2,1) = 0.85; 95% CI, 0.723–0.922, *p* = 9.385 × 10^-12^). Our findings show that dynamic MRI can provide reviewable, reproducible visualization of tendon motion at the level of the lateral malleolus. From a clinical perspective, the potential value of this approach is to extend ankle MRI beyond static morphology toward functional assessment of tendon behavior during motion. Dynamic imaging may be particularly relevant when symptoms suggest peroneal instability but static MRI is equivocal, or when ultrasound findings are discordant or difficult to reproduce across operators. While ultrasound remains an excellent first-line dynamic tool, dynamic MRI offers a standardized acquisition within the MRI examination and provides a record that can be reviewed retrospectively by other radiologists and by the treating clinician, which may support multidisciplinary decision-making. At this feasibility stage, we do not claim improved diagnostic accuracy; rather, we provide the technical and measurement groundwork needed for future patient studies assessing diagnostic yield and impact on management.

Tendon stability is primarily a clinical diagnosis; however, functional imaging can be very helpful. Tracking the relative position of the tendons is a prerequisite for assessing it. Importantly, centroid-to-centroid distance should be interpreted as a research metric reflecting inter-tendon spacing and translation rather than as a validated diagnostic threshold for instability. By showing that dynamic MRI provides reproducible, time-resolved measurements of tendon motion, this pilot study establishes a foundation for future studies linking motion trajectories to clinically defined instability in symptomatic cohorts.

Whether earlier confirmation of abnormal mobility influences referral pathways or conservative management should be evaluated in future studies in symptomatic cohorts. Future studies should evaluate dynamic MRI in symptomatic cohorts, ideally against ultrasound and clinical/surgical reference standards, to determine whether motion metrics, including excursion patterns, can define clinically meaningful thresholds.

Dynamic MRI has been recognized as a promising technique for assessing real-time joint function, and may complement static MRI in evaluating motion-dependent pathologies. Systematic reviews affirm that dynamic MRI provides valid and reproducible data for larger joint motion analysis, although protocols remain heterogeneous. However, dynamic MRI is not widespread in musculoskeletal diagnostics, mainly due to technical challenges and the difficulty in acquiring consistently high-quality images. Various dynamic MRI techniques exist and have been applied inconsistently across centers, limiting reproducibility and hindering comparison between studies. Our study demonstrates that, with an optimized protocol and setup, reliable results are achievable, laying the groundwork for real-time evaluation of ankle structures and, potentially, other joints. In previous studies, images generated during dynamic MRI have not always demonstrated high quality across different structures. One possible explanation is that much of the musculoskeletal work has focused on the motion of bones, primarily the knee joint, and assessing bony kinematics does not demand the same level of precision as the evaluation of small tendons or retinaculum. Reflecting this issue, the ankle joint has been evaluated only in two studies without a focus on the peroneal tendons. Moreover, studies of other joints have shown that real-time dynamic MRI acquisition protocols can suffer from artifacts or loss of fidelity if the joint is not positioned centrally within the bore or the coil shifts during movement. The imaging protocol in our study makes it possible to achieve high-quality dynamic examinations that allow evaluation of small stabilizing structures. The protocol minimizes motion artifacts with the use of a sandbag and carefully placed foam supports to stabilize the ankle while permitting a full range of dorsiflexion and plantarflexion. Static multi-position imaging samples discrete end-points and may not reflect physiological conditions during active motion, including active muscle activation and tendon loading. Dynamic imaging captures continuous motion and provides time-resolved trajectories, which may reveal transient, intermittent events not captured by a limited set of static positions.

Allowing ankle movement required a less tightly fitted coil setup than in a conventional static examination, likely resulting in some local SNR penalty. Because no directly comparable reference acquisition with tighter coil positioning was performed, this effect could not be reliably quantified from our data. Prior work supports the principle that more conformal coil positioning improves local signal reception, but dynamic MRI also requires sufficient freedom of movement within the coil and careful setup to permit joint motion during acquisition [6, 14, 15]. In our study, this limitation did not appear to compromise the intended functional assessment and is likely more relevant for applications requiring maximal local SNR and fine structural detail.

This study has several limitations. Single-center feasibility in asymptomatic volunteers limits generalizability and does not establish diagnostic performance. Because we included only healthy asymptomatic volunteers, the feasibility in symptomatic patients remains unknown. Pain and limited range of motion may restrict active movement and reduce the feasibility of the proposed dynamic protocol; this should be assessed in future patient studies. Quantification used single-rater manual segmentation; inter-rater and test–retest reproducibility were not assessed. In addition, fully manual segmentation should be regarded as a research workflow and is unlikely to be practical for routine clinical use. Broader clinical implementation will likely require automated or AI-assisted segmentation with limited manual quality control. Motion was self-paced and imaging confined to a single axial level (10 mm), so through-plane and partial-volume effects are possible; the frame rate of 0.59 s/frame may miss very rapid motion. The ankle joint angles were not directly measured during dynamic acquisition. Motion comparability was based on standardized instructions, practice runs, and cycle normalization rather than identical absolute dorsiflexion/plantarflexion angles. The 10-mm slice thickness represented a deliberate trade-off to preserve SNR and 0.59-s temporal resolution with balanced fast field echo and compressed sensitivity encoding; however, it increased through-plane partial-volume effects and sensitivity to through-plane motion, limiting detection and localization of small focal pathology, which was therefore assessed on the static high-resolution sequences. All scans used one 3-T system/coil from a single vendor. Objective SNR or contrast-to-noise ratio quantification was not considered reliable in this dynamic setting. Although subtraction-based methods are preferable for phased-array imaging, they require two closely matched acquisitions, which was not feasible during active, self-paced motion because repeated scans could differ in motion state, position, slice location, and through-plane displacement. Image quality was therefore assessed by structured reader evaluation. To improve consistency, we mitigated variability with standardized instructions, practice runs, real-time monitoring, ankle stabilization, and predefined criteria with agreement analysis. Future studies should include symptomatic patients, multi-reader/multi-center design, gated or metronome-cued motion, broader coverage or 3D imaging with higher temporal resolution, as well as comparison with clinical/surgical reference standards to define abnormal excursion thresholds.

Dynamic 3-T MRI may, in the future, serve as a complementary method for assessing peroneal tendon motion during active dorsiflexion and plantarflexion. Although ultrasound remains the primary dynamic imaging modality, MRI-based motion metrics could be useful in selected problem-solving scenarios, particularly in patients with motion-provoked symptoms or discordant clinical and imaging findings. They may also provide a framework for future studies on severity grading and pre- *versus* post-intervention assessment. Before routine clinical use can be considered, studies in symptomatic cohorts are required to establish reproducibility, diagnostic performance, incremental value over ultrasound and conventional MRI, and potential effects on clinical management and cost-effectiveness. At present, these findings should be considered hypothesis-generating and require confirmation in adequately powered studies, ideally with correlation to clinical findings and, where available, ultrasound and surgical reference standards.

In conclusion, dynamic ankle 3-T MRI feasibly depicts peroneal tendon motion during active movement with diagnostically acceptable image quality. Reader agreement was moderate-to-substantial, and centroid-distance measurements were reliable. These findings support dynamic MRI as a reproducible method to visualize and quantify peroneal tendon motion.

## Supplementary information

**Additional File 1 :** Supplementary material 2. Additional figures and videos. **Film 1**. Peroneal tendons (arrow) examined on 3-T MRI. Right ankle. **Film 2**. Peroneal tendons (arrow) examined on 3-T MRI. Left ankle. **Figure S1**. Study flow diagram of inclusion and analysis steps. **Figure S2**. Distribution of distances between the centroids of the peroneus longus and peroneus brevis tendons for each volunteer. The upper panel shows violin plots with overlaid individual frame measurements. The lower panel shows corresponding box plots indicating the median, interquartile range, and variability. **Figure S3**. Three-dimensional ribbon-surface visualizations of tendon motion characteristics across time and for each volunteer. (**a**) Absolute inter-tendon distance (mm) across normalized time. (**b**) Speed, defined as the first derivative of distance with respect to frame number (mm/frame). (**c**) Curvature, defined as the second derivative of distance with respect to frame number (mm/frame²), highlighting acceleration/deceleration and abrupt changes in motion. The X-axis shows normalized time (0–1), and the Y-axis indexes the volunteers (1–10). Figure 3A-C is also included as separate Electronic Supplementary Material.

Supplementary Video 1

Supplementary Video 2

## Data Availability

The datasets generated and analyzed during the current study, as well as custom scripts, are available as supplementary material and from the corresponding author upon reasonable request.

## Abbreviations

AC2: Gwet’s agreement coefficient 2
CI: Confidence interval
ICC: Intraclass correlation coefficient
PB: Peroneus brevis
PL: Peroneus longus
SD: Standard deviation
SNR: Signal-to-noise ratio

## References

[1] DiGiovanni BF, Fraga CJ, Cohen BE, Shereff MJ (2000) Associated injuries found in chronic lateral ankle instability. Foot Ankle Int 21:809–815. 10.1177/107110070002101003 11128010 10.1177/107110070002101003

[2] Karlsson J, Wiger P (2002) Longitudinal split of the peroneus brevis tendon and lateral ankle instability: treatment of concomitant lesions. J Athl Train 37:463–466 12937568 PMC164378

[3] Bokwa-Dabrowska K, Mocanu D, Alexiev A, Helander KN, Szaro P (2024) Peroneus brevis split rupture is underreported on magnetic resonance imaging of the ankle in patients with chronic lateral ankle pain. Eur J Radiol Open 13:100591. 10.1016/j.ejro.2024.100591 39131949 10.1016/j.ejro.2024.100591 PMC11314861

[4] Hosseinian SHS, Aminzadeh B, Rezaeian A, Jarahi L, Naeini AK, Jangjui P (2022) Diagnostic value of ultrasound in ankle sprain. J Foot Ankle Surg 61:305–309. 10.1053/j.jfas.2021.08.008 34565666 10.1053/j.jfas.2021.08.008

[5] Alavekios DA, Dionysian E, Sodl J, Contreras R, Cho Y, Yian EH (2013) Longitudinal analysis of effects of operator experience on accuracy for ultrasound detection of supraspinatus tears. J Shoulder Elbow Surg 22:375–380. 10.1016/j.jse.2012.09.017 23312821 10.1016/j.jse.2012.09.017

[6] Garetier M, Borotikar B, Makki K, Brochard S, Rousseau F, Ben Salem D (2020) Dynamic MRI for articulating joint evaluation on 1.5 T and 3.0 T scanners: setup, protocols, and real-time sequences. Insights Imaging 11:66. 10.1186/s13244-020-00868-5 32430739 10.1186/s13244-020-00868-5 PMC7237553

[7] Neustadter J, Raikin SM, Nazarian LN (2004) Dynamic sonographic evaluation of peroneal tendon subluxation. AJR Am J Roentgenol 183:985–988. 10.2214/ajr.183.4.1830985 15385290 10.2214/ajr.183.4.1830985

[8] Melville DM, Taljanovic MS, Gimber LH et al (2024) Comparison of ultrasound and MRI with intraoperative findings in the diagnosis of peroneal tendinopathy, tears, and subluxation. J Clin Med 13:740. 10.3390/jcm13030740 38337434 10.3390/jcm13030740 PMC10856550

[9] Pesquer L, Guillo S, Poussange N, Pele E, Meyer P, Dallaudière B (2016) Dynamic ultrasound of peroneal tendon instability. Br J Radiol 89:20150958. 10.1259/bjr.20150958 26943704 10.1259/bjr.20150958 PMC5257307

[10] Sullivan GM, Artino AR Jr (2013) Analyzing and interpreting data from Likert-type scales. J Grad Med Educ 5:541–542. 10.4300/JGME-5-4-18 24454995 10.4300/JGME-5-4-18 PMC3886444

[11] Russell CJ, Bobko P (1992) Moderated regression analysis and Likert scales: too coarse for comfort. J Appl Psychol 77:336–342. 10.1037/0021-9010.77.3.336 1601825 10.1037/0021-9010.77.3.336

[12] Koo TK, Li MY (2016) A guideline of selecting and reporting intraclass correlation coefficients for reliability research. J Chiropr Med 15:155–163. 10.1016/j.jcm.2016.02.012 27330520 10.1016/j.jcm.2016.02.012 PMC4913118

[13] Landis JR, Koch GG (1977) The measurement of observer agreement for categorical data. Biometrics 33:159–174. 10.2307/2529310 843571

[14] Nohava L, Obermann M, Frass-Kriegl R, Soanca O, Laistler E (2025) A modular system of flexible receive-only coil arrays for 3 T magnetic resonance imaging. Z Med Phys 35:193–203. 10.1016/j.zemedi.2023.05.002 37258388 10.1016/j.zemedi.2023.05.002 PMC12166917

[15] Ohno N, Miyati T, Niwa Y et al (2018) Novel practical SNR determination method for MRI using double echo with longest second echo time (DELSET). Br J Radiol 91:20170652. 10.1259/bjr.20170652 29565674 10.1259/bjr.20170652 PMC6223277

