# Supplementary material for: Real-time 3-T MRI of peroneal tendon motion during active ankle movement: feasibility and image quality
Source: Eur Radiol Exp. 2026 Jun 25;10:99. doi: 10.1186/s41747-026-00762-7 (PMC13305081; doi:10.1186/s41747-026-00762-7)
Supplement: Supplementary file 1 — Additional File 1 : Supplementary material 2. Additional figures and videos. Film 1. Peroneal tendons (arrow) examined on 3-T MRI. Right ankle. Film 2. Peroneal tendons (arrow) examined on 3-T MRI. Left ankle. Figure S1. Study flow diagram of inclusion and analysis steps. Figure S2. Distribution of distances between the centroids of the peroneus longus and peroneus brevis tendons for each volunteer. The upper panel shows violin plots with overlaid individual frame measurements. The lower panel shows corresponding box plots indicating the median, interquartile range, and variability. Figure S3. Three-dimensional ribbon-surface visualizations of tendon motion characteristics across time and for each volunteer. (a) Absolute inter-tendon distance (mm) across normalized time. (b) Speed, defined as the first derivative of distance with respect to frame number (mm/frame). (c) Curvature, defined as the second derivative of distance with respect to frame number (mm/frame²), highlighting acceleration/deceleration and abrupt changes in motion. The X-axis shows normalized time (0–1), and the Y-axis indexes the volunteers (1–10). Figure 3A-C is also included as separate Electronic Supplementary Material. [file 41747_2026_762_MOESM1_ESM.pdf]

Real-time 3T MRI of peroneal tendon motion during active ankle movement: feasibility and image quality

ELECTRONIC SUPPLEMENTARY MATERIAL

Supplementary material 1

Code A

```
// ImageJ macro: manual segmentation of fibula, peroneus longus and peroneus
brevis on DICOM images
// For each DICOM image in the input directory, the user:
//   1) Draws ROIs for Fibula, Peroneus Longus, and Peroneus Brevis.
// The macro assumes that the DICOM metadata already contains pixel spacing,
// so area measurements are reported in mm^2 (if the unit is mm).
// The macro saves areas (mm^2) to a CSV file and ROI coordinates to TXT files.

// === User-configurable paths ===
inputDir       = "/path/to/input/dicom/";                    // folder with DICOM images (.dcm)
outputFile     = "/path/to/output/results_summary.csv";       // CSV summary of cross-
sectional areas
coordinatesDir = "/path/to/output/coordinates_txt/";          // folder for ROI coordinate
TXT files

// === Initialize output files ===
File.delete(outputFile);
File.append("Filename,Fibula (mm^2),Peroneus Longus (mm^2),Peroneus Brevis
(mm^2)\n", outputFile);

if (!File.exists(coordinatesDir)) {
    File.makeDirectory(coordinatesDir);
}

// === Load files ===
list = getFileList(inputDir);

// === Process all DICOM files ===
firstImageChecked = false;

for (i = 0; i < list.length; i++) {

    // Adjust extension filter if needed (e.g. other DICOM extensions)
    if (endsWith(list[i], ".dcm")) {

        open(inputDir + list[i]);
```

```
        // Optional: verify that the spatial unit is mm
        if (!firstImageChecked) {
            getPixelSize(unit, pw, ph, depth);
            if (unit != "mm") {
                showMessage("Calibration Warning",
                    "DICOM pixel unit is not mm. Current unit: " + unit);
            }
            firstImageChecked = true;
        }

        // === FIBULA ===
        waitForUser("Draw FIBULA on " + list[i]);
        fibula_area = "NaN";
        if (selectionType() != -1) {
            run("Measure");
            fibula_area = getResult("Area", nResults - 1);
            saveCoordinatesToTXT("Fibula", list[i], coordinatesDir);
        }

        // === PERONEUS LONGUS ===
        waitForUser("Draw PERONEUS LONGUS on " + list[i]);
        longus_area = "NaN";
        if (selectionType() != -1) {
            run("Measure");
            longus_area = getResult("Area", nResults - 1);
            saveCoordinatesToTXT("Peroneus_Longus", list[i], coordinatesDir);
        }

        // === PERONEUS BREVIS ===
        waitForUser("Draw PERONEUS BREVIS on " + list[i]);
        brevis_area = "NaN";
        if (selectionType() != -1) {
            run("Measure");
            brevis_area = getResult("Area", nResults - 1);
            saveCoordinatesToTXT("Peroneus_Brevis", list[i], coordinatesDir);
        }

        // === Save results for this image ===
        File.append(stripExtension(list[i]) + "," +
                    fibula_area + "," +
                    longus_area + "," +
                    brevis_area + "\n", outputFile);

        run("Clear Results");
        close();
    }
}

// === Function: Save ROI coordinates to TXT ===
function saveCoordinatesToTXT(structure, fileName, coordinatesDir) {
```

```
    getSelectionCoordinates(x, y);
    output = "Coordinates for " + structure + " in file: " + fileName + "\n";

    if (x.length > 0) {
        for (j = 0; j < x.length; j++) {
            output += "Point " + (j + 1) + ": X=" + x[j] + ", Y=" + y[j] + "\n";
        }
    } else {
        output += "No coordinates found.\n";
    }

    txtFile = coordinatesDir + stripExtension(fileName) + "_" + structure + ".txt";
    File.saveString(output, txtFile);
}

// === Function: Strip file extension ===
function stripExtension(fileName) {
    dotIndex = lastIndexOf(fileName, ".");
    return substring(fileName, 0, dotIndex);
}
```

Code B

```
# R script: centroid extraction and distances between fibula, peroneus longus and peroneus brevis
#
# Input:
#   TXT files exported from the ImageJ macro (Appendix A), each containing lines:
#       "Point i: X=<value>, Y=<value>"
#   The file names encode patient and slice (e.g. PatientID.SliceID_Structure.txt).
#
# Output:
#   A CSV file with:
#     - centroids (centroid_x, centroid_y) for each structure
#     - pairwise distances between structures in pixels and mm
#
# Note:
#   'scale_pixels_per_mm' should be set according to the image calibration
#   (e.g. from DICOM pixel spacing).

library(dplyr)
library(stringr)
library(readr)
library(tidyr)

# === User-configurable parameters ===

# Folder containing TXT files exported from ImageJ
input_folder <- "/path/to/coordinates_txt/"
```

```
# Output CSV file with centroids and distances
output_file  <- "/path/to/output/centroid_distances_summary.csv"

# Number of pixels per millimetre (replace with your actual value)
scale_pixels_per_mm <- 18.4

# === Read all TXT files ===
txt_files <- list.files(input_folder, pattern = "\\.txt$", full.names = TRUE)

# Function to extract centroid from a single TXT file
extract_centroid <- function(file) {
  lines  <- readLines(file)
  coords <- lines[grepl("X=", lines)]

  x_vals <- as.numeric(str_extract(coords, "(?<=X=)\\d+\\.*\\d*"))
  y_vals <- as.numeric(str_extract(coords, "(?<=Y=)\\d+\\.*\\d*"))

  tibble(
    PatientID = str_extract(basename(file), "^.*?(?=\\.\\d{4})"),
    SliceID   = str_extract(basename(file), "(?<=\\.)\\d{4}"),
    Structure = case_when(
      str_detect(file, "Fibula")          ~ "Fibula",
      str_detect(file, "Peroneus_Longus") ~ "PL",
      str_detect(file, "Peroneus_Brevis") ~ "PB",
      TRUE                                ~ "Unknown"
    ),
    centroid_x = mean(x_vals, na.rm = TRUE),
    centroid_y = mean(y_vals, na.rm = TRUE)
  )
}

# Extract centroids from all files
centroids <- bind_rows(lapply(txt_files, extract_centroid))

# Reshape to one row per patient and slice
centroids_wide <- centroids %>%
  pivot_wider(
    id_cols     = c(PatientID, SliceID),
    names_from  = Structure,
    values_from = c(centroid_x, centroid_y),
    names_glue  = "{.value}_{Structure}"
  )

# Compute pairwise distances (in pixels and mm)
centroids_wide <- centroids_wide %>%
  mutate(
    dist_Fibula_PL = sqrt((centroid_x_Fibula - centroid_x_PL)^2 +
                          (centroid_y_Fibula - centroid_y_PL)^2),
    dist_Fibula_PB = sqrt((centroid_x_Fibula - centroid_x_PB)^2 +
```

```
                      (centroid_y_Fibula - centroid_y_PB)^2),
    dist_PL_PB     = sqrt((centroid_x_PL     - centroid_x_PB)^2 +
                      (centroid_y_PL     - centroid_y_PB)^2),

    dist_Fibula_PL_mm = dist_Fibula_PL / scale_pixels_per_mm,
    dist_Fibula_PB_mm = dist_Fibula_PB / scale_pixels_per_mm,
    dist_PL_PB_mm     = dist_PL_PB     / scale_pixels_per_mm
  )

# Final result table
final_result <- centroids_wide %>%
  mutate(Filename = paste(PatientID, SliceID, sep = ".")) %>%
  select(
    Filename,
    starts_with("centroid_x"),
    starts_with("centroid_y"),
    starts_with("dist_")
  )

# Save to CSV
write_csv(final_result, output_file)

cat("Distances calculated and saved to:\n", output_file, "\n")
```

Supplementary material 2. Additional figures and videos.
Film 1. Peroneal tendons (arrow) examined on 3T MRI. Right ankle.
Film 2. Peroneal tendons (arrow) examined on 3T MRI. Left ankle.

Figure S1. Study flow diagram of inclusion and analysis steps.

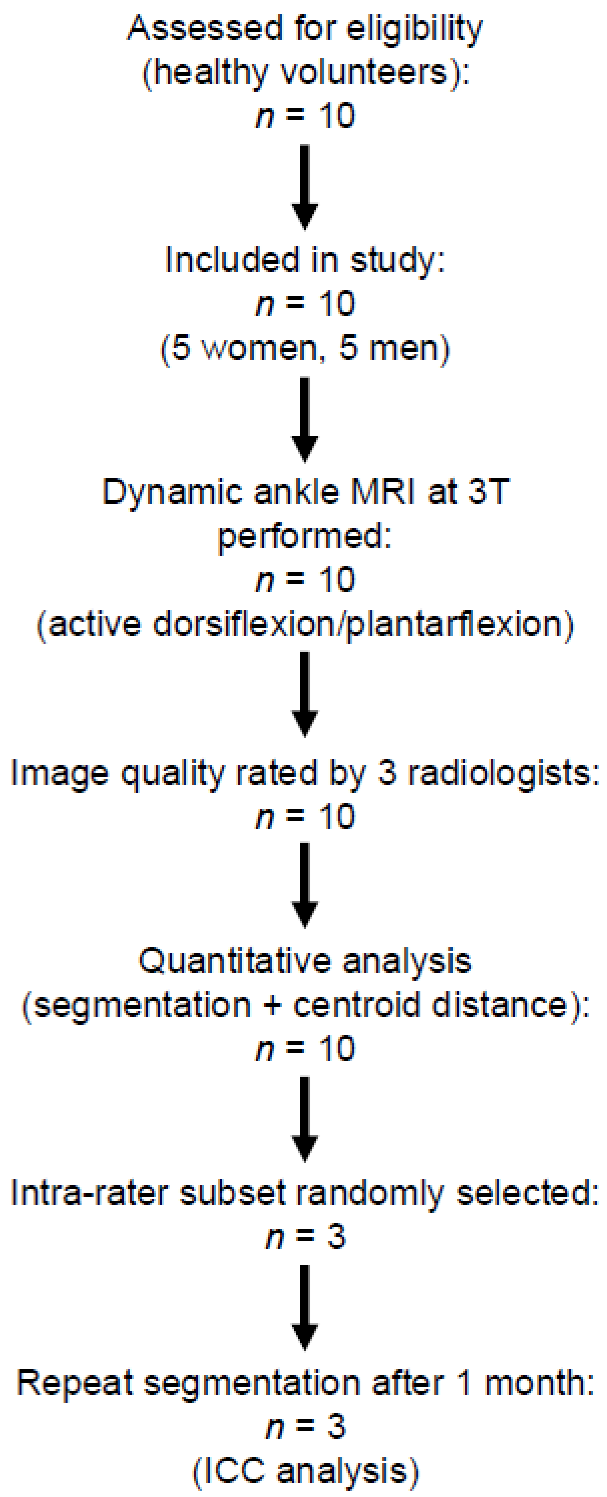

Figure S2. Distribution of distances between the centroids of the peroneus longus and peroneus brevis tendons for each volunteer. The upper panel shows violin plots with overlaid individual frame measurements. The lower panel shows corresponding box plots indicating the median, interquartile range, and variability.

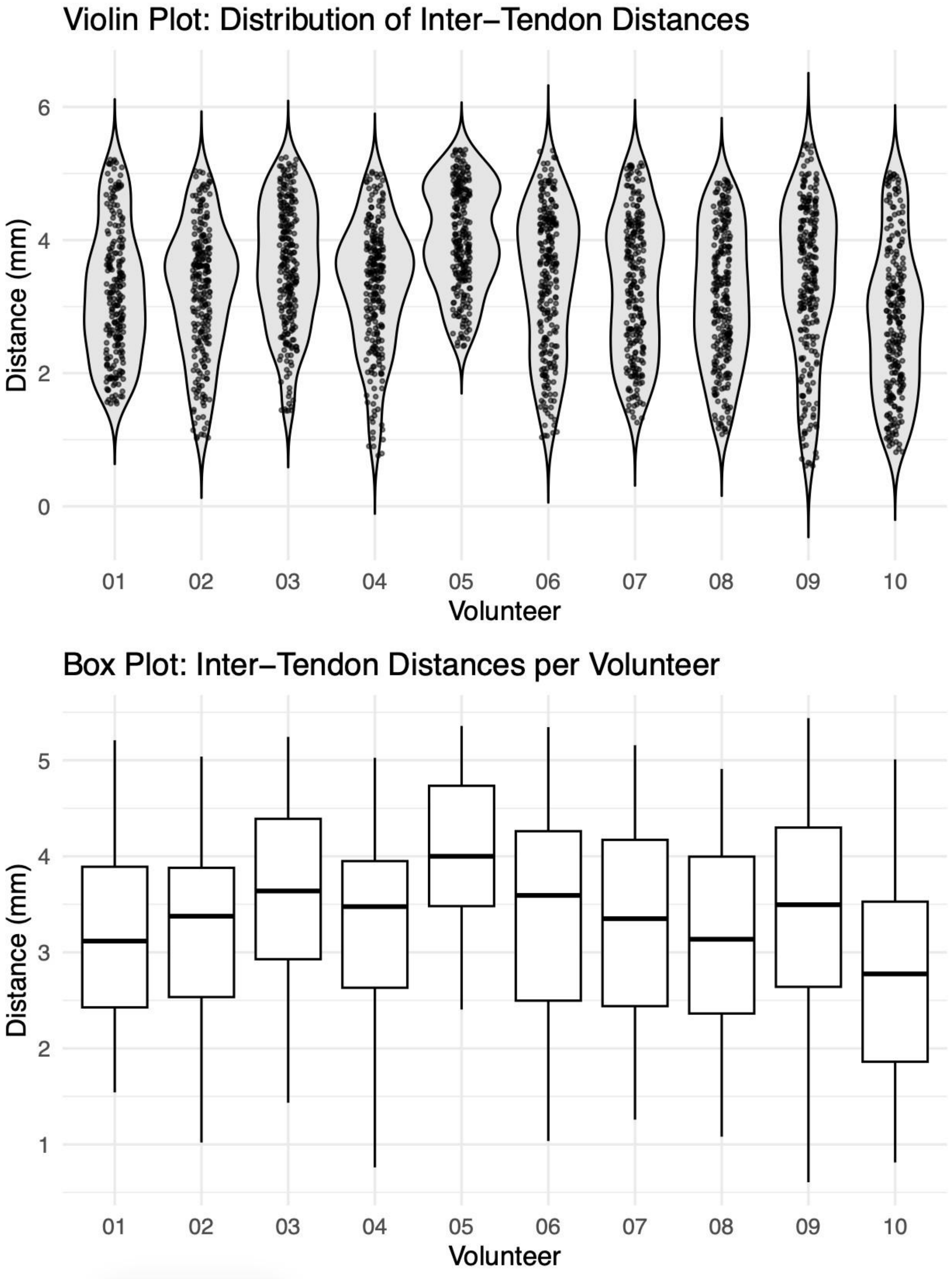

Figure S3. Three-dimensional ribbon-surface visualizations of tendon motion characteristics across time and for each volunteer.
(a) Absolute inter-tendon distance (mm) across normalized time.
(b) Speed, defined as the first derivative of distance with respect to frame number (mm/frame).
(c) Curvature, defined as the second derivative of distance with respect to frame number (mm/frame²), highlighting acceleration/deceleration and abrupt changes in motion. The X-axis shows normalized time (0–1), and the Y-axis indexes the volunteers (1–10).

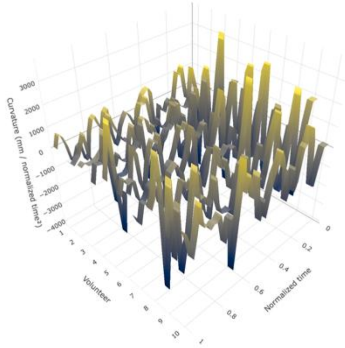

a

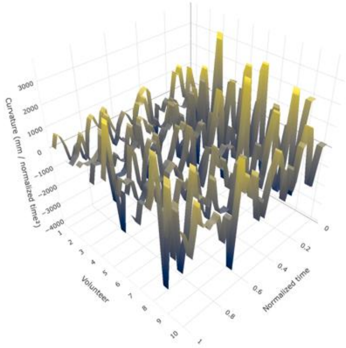

b

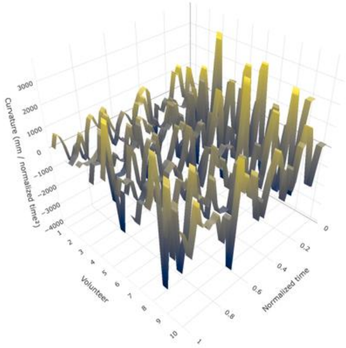

c

Figure 3A-C is also included as separate Electronic Supplementary Material
